# Methylphenidate off-label use and safety

**DOI:** 10.1186/2193-1801-3-286

**Published:** 2014-06-07

**Authors:** Thierry Trenque, Emmanuelle Herlem, Malak Abou Taam, Moustapha Drame

**Affiliations:** Regional Centre for Pharmacovigilance and Pharmacoepidemiology, Reims University Hospitals, Avenue du General Koenig, 51092, Reims, France; Faculty of Medicine, University of Reims Champagne-Ardenne, EA 3797, F-51095 Reims, France; Department of Research and Innovations, Reims University Hospitals, Avenue du General Koenig, 51092 Reims, France

**Keywords:** Methylphenidate, Off-label use

## Abstract

**Introduction:**

Methylphenidate is a piperidine derivative structurally and pharmacologically similar to amphetamine. Methylphenidate is indicated for Attention Deficit Hyperactivity Disorder (ADHD) in children aged 6 years of age and over when remedial measures alone prove insufficient. In adults, its indication, except in narcolepsy, is not defined. Methylphenidate received regulatory approval almost sixty years ago with a first registration in Switzerland in October 1954.

**Objective:**

To evaluate the off-label use of methylphenidate and its characteristics from a database of spontaneous reports.

**Methods:**

This study analysed data from the French Pharmacovigilance Database of adverse drug reactions spontaneously reported by health professionals from 1985 to December 2011. Off-label use was evaluated according to age.

**Results:**

In the French Pharmacovigilance database, 181 cases of adverse drug reactions were reported with methylphenidate. Neuropsychiatric effects were the most frequent adverse event reported (41%) followed by cardiovascular and cutaneous side effects (14%). 143 reports concerned children (113 boys, 30 girls, mean age 10.6 ± 3.3 years) of which 46 (30%) were off-label uses. There were 38 adults (20 men, 18 women), of which 32 (88%) off-label use. In adults, methylphenidate was prescribed for depression, and this practice was associated with serious adverse events of drug dependence, overdose and suicide attempt. Overall, off-label use was detected in 43% (78/181) of all cases reported.

**Conclusion:**

More than 40% of the patients with drug reactions received methylphenidate for off-label indications. Additional long-term exposures and independent clinical studies are necessary to establish the long-term profile safety of methylphenidate.

## Introduction

Methylphenidate hydrochloride [(dl-threo-methyl-2-phenyl-2-(2-piperidyl) acetate] is a central nervous system stimulant derived from piperidine that is structurally similar to amphetamine, and acts as a norepinephrine - dopamine reuptake inhibitor. It increases dopaminergic neurotransmission, particularly at the striatal and frontal levels, by inhibiting presynaptic dopamine transporters. By blocking reuptake of noradrenaline and dopamine at the level of the presynaptic neuron, it increases the release of these monoamines at the level of the synaptic cleft. The effect of methyphenidate is mediated by blocking the Dopamine Transporter (DAT), which increases the synaptic concentration of dopamine (Volkow et al. 2002), known to be a key neuromodulating agent. The ultimate result is increased attention, motor function and memory. Despite the extensive literature investigating methylphenidate, its mode of action and its effects, the exact mechanism of its therapeutic action remains unclear.

Methylphenidate is indicated for the treatment of Attention Deficit Hyperactivity Disorder (ADHD), one of the most common psychiatric disorders diagnosed in children (Scahill and Schwab-Stone 2000). It was first registered under the brand name Ritalin^®^ in Switzerland almost sixty years ago.

In France, it is available in both immediate release (Ritaline^®^ 10 mg), and sustained release forms (Ritaline LP^®^, Quasym^®^ and Concerta^®^). The immediate release form is also indicated for adults with narcolepsy. For all forms of methylphenidate, it is recommended not to exceed 60 mg per day. Methylphenidate is indicated as a part of a comprehensive treatment programme for ADHD in children aged 6 years and over, when remedial measures alone prove insufficient. Treatment must be under the supervision of a specialist in childhood behavioural disorders, and the diagnosis should be made according to the Diagnostic and Statistical Manual of Mental Disorders (DSM-IV) (American Psychiatric Association 2000) criteria or the International Classification of Diseases release 10 (ICD10). A comprehensive treatment programme typically includes psychological, educational, and social measures.

In France, methylphenidate is listed as a narcotic and has restrictive conditions of prescription and dispensing. The initial prescription must be signed by a hospital neurologist, psychiatrist or paediatrician on a specific form for scheduled drugs, and prescription duration is limited to twenty-eight days. The prescription can be renewed by any other physician for a maximum of one year, but the dose and quantity prescribed by the initial hospital-based physician cannot be changed. Moreover, pharmacies are only allowed to dispense methylphenidate on presentation of the two prescriptions, namely the initial hospital prescription and the renewal prescription. A contract of care is established between the pharmacist, the practitioner, and the patient. The names of the three individuals are listed on the renewal prescription, and the diagnosis must be re-evaluated each year by the specialist.

Despite the limited indications, and the stringent conditions of its prescription and dispensing, off-label use of methylphenidate exists. The guidelines of the European Medicines Agency define off-label use as “medicinal product intentionally used for a medical purpose not in accordance with the authorised product information” (European Medicines Agency 2012). In this context, we aimed to evaluate the off-label use of methylphenidate, and describe the features of off-label use from a database of spontaneous reports for methylphenidate in France.

## Methods

This study used data from the French National Pharmacovigilance Database of all Adverse Drug Reactions (ADRs) spontaneously reported with commercially approved drugs in France. This database was established in 1985 to register all ADRs spontaneously reported by health professionals to the French Pharmacovigilance System, but not those reported to manufacturers. The national database includes data from 31 regional pharmacovigilance centres. Reports are reviewed by medically qualified personnel in the regional centres before being entered into the national database. ADRs are coded according to the Medical Dictionary for Regulatory Activities (MedDRA^®^, version 12.1) (Brown and Sexson 1988).

Seriousness of the reaction is also recorded in the Pharmacovigilance database: ADRs are considered serious when they result in death, are life-threatening, require patient hospitalisation or prolongation of existing hospitalisation, or any reaction that results in persistent or significant disability or incapacity, congenital anomaly or birth defect. All other ADRs are considered non-serious (European Medicines Agency 2012).

All cases reported between 1 January 1985 and 31 December 2011 and citing methylphenidate (in any of its forms) were selected. All cases were assessed from the computerised data and reviewed by two pharmacovigilance specialists (TT, EH) to determine whether the use was off-label. The criteria for classifying the use as off-label were as follows:

- all patients below six years of age treated with methylphenidate were considered as off-label use,
- all adults (18 years and above) treated with methylphenidate for indications other than narcolepsy were considered as off-label use,
- for narcolepsy, only the immediate-release form (Ritaline^®^) is approved for use in adults.
- intentional drug misuse

For each case reported, we recorded the patient’s characteristics (age, gender, and underlying disease), prescription data (dose, dosage, indication) and the characteristics of the ADR (clinical symptoms, seriousness, mean onset delay, course, outcome). The data in the French Pharmacovigilance Database are anonymous. The study got the approval of the Institutional Review Board of the University Hospitals of Reims.

## Results

From 1 January 1985 to 31 December 2011, in the French National Pharmacovigilance database, 181 spontaneous reports were collected citing methylphenidate as the culprit drug. Among these, 78 (43%) were considered to be off-label uses of the drug.

**Figure 1:**
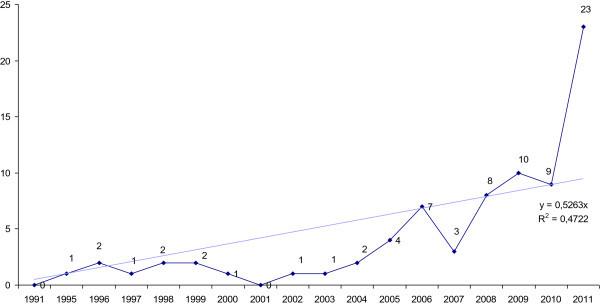
**Profile of the number of spontaneous reports of adverse effects occurring during off-label use of methylphenidate in the French National Pharmacovigilance Database from 1991 to 2011.**

Overall, 143 children were involved (113 boys, 30 girls), mean age 10 years, of which 46 (32%) were off-label use. In children, when used in accordance with its indications, serious adverse effects represented 31.3% of all spontaneous ADRs, and 43% of the spontaneous reports in the context of off-label use.There were 38 spontaneous reports in adults (20 men, 18 women) of which 32 (84%) were considered as off-label use. Reports in adults first appeared in 2005 and now represent 27% of the spontaneous notifications. In adults, when used in accordance with its indications, serious adverse effects represented 33% of all spontaneous reports, and 52% of the spontaneous reports in the context of off-label use. The number of spontaneous reports with methylphenidate is increasing regularly, and doubled over the year 2011 (Figure 1). By 2011, off-label use notifications represented half of the total spontaneous notifications reported to the national pharmacovigilance database with methylphenidate.

For patients aged <6 years, the main indications for prescribing methylphenidate were ADHD, autism and psychomotor activity. In the 6–18 year age group, the off-label indications for methylphenidate prescription were: behavioural disorders (38%), narcolepsy (Concerta^®^) (13%), instability (19%), autism (13%) and intellectual disability (8.8%).

Off-label uses of the drug, with presence of a contraindication, were observed in 9 cases (i.e. 20% of off-label use in children) (child aged <6 years, n = 6; prior history of epilepsy, n = 3).

In adults, the main indications for prescription of methylphenidate were: ADHD (n = 5), representing 13% of spontaneous reports in adults; narcolepsy-hypersomnia (n = 5, 13%); depression (n = 2, 5%) and Parkinson’s disease (n = 2, 5%).

Neuropsychiatric effects were the most frequent event reported, followed by cardiovascular and cutaneous side effects, both reported in 13.3% of cases.

Muscular effects were reported in 7.2%, and misuse represented 5.5% of ADRs. Lesions, intoxication and procedural complications represented 4.4% of cases; intestinal, hepatobiliary and haematological effects in 3%; urological, dental or ear-nose-throat effects, as well as biological modifications were rare (<1%).

## Discussion

In our study, we investigated prescriptions of methylphenidate outside of the described product characteristics, using data from the French National Pharmacovigilance Database. Despite very restrictive conditions for its prescription in France, over 40% of the spontaneous ADRs with methylphenidate could be considered as off-label use (age <6 years, or prescription in adults outside of the only approved indication, namely narcolepsy). The ADRs observed were more often classed as serious when the use was off-label.

In our study, we chose to take into account only the criteria based on age and indication, as this information is generally always recorded in pharmacovigilance databases. Thus, it is likely that the high rate of off-label use observed is actually an under-estimation of the true rate.

Possible sources for identifying off-label prescriptions are manifold, but unfortunately, not well structured. First among these sources are the pharmacovigilance databases, but other sources are also available, such as hospital databases, sales information or marketing studies, and data on reimbursement of medications through the social security system. The data from our study were taken only from the French National Pharmacovigilance Database. Spontaneous reporting systems are associated with under-reporting. The tendency of physicians to under-report is heightened by the fear of revealing an off-label (unapproved) use of the drug (Inman 1996).

The motives for prescription of methylphenidate in adults varied widely in this study, with prescription for ADHD representing only 1/6 of all reports. The use of methylphenidate in adults is not codified, and there are currently almost 300 studies ongoing with this drug (http://clinicaltrials.gov, accessed 23/08/2013).

Our data are in line with the few studies of methylphenidate prescription reported to date (Frauger et al. 2011; Knellwolf et al. 2008; Wilens et al. 2008).

Among psychiatric drugs, off-label use is reportedly as high as 31% (Radley et al. 2006).

Identifying off-label use is difficult, as this is not a specific item in databases that record ADRs. In addition, off-label use is legal in many countries, including the USA, and thus, in such cases, there would be no particular justification for noting whether use was on- or off-label. In France, off-label use is wholly under the responsibility of the prescriber. Off-label prescribing is not uncommon, and one third of chemotherapies used to fight cancer are reportedly prescribed “off-label” (Conti et al. 2013). Similarly, in the United States, one report estimated that over 20% of prescriptions were to treat diseases or conditions in non-FDA approved indications (Radley et al. 2006). In theory, the pharmaceutical industry cannot encourage or promote off-label use of its products. However, in December 2012, an American circuit court considered that in accordance with the first Amendment to the Constitution of the United States of America, every individual has the right to freedom of speech, and off-label marketing was considered to fall into this category (off-label Drug Marketing is “Free Speech” Court Rules - ABC News Retrieved 10 December 2012).

Methylphenidate is approved for the treatment of ADHD. However, the diagnosis of ADHD is controversial due to the existence of two different classification systems, namely the International Classification of Mental and Behavioural Disorders 10th revision (ICD10), and the Diagnosis and Statistical Manual Mental Disorders 4th edition (DSM-IV), used as the reference in the labelling. The ICD10 is published by the World Health Organization to track morbidity and mortality statistics for all diseases. The DSM-IV is developed by American physicians and used as a classification for mental disorders.

These two classifications differ considerably in the diagnostic criteria used to qualify ADHD, with reported agreement of only 68% (Andrews et al. 1999). For the diagnosis of ADHD, the ICD10 classification is more restrictive in four ways. Firstly, it requires that symptoms appear before the age of 6 years. Second, it prohibits the diagnosis when the patient has anxiety, mood disorders, or schizophrenia, which is a restriction not imposed by the DSM-IV classification. Third, the ICD10 diagnosis requires that the inattention be directly observed by the clinician. Finally, the DSM-IV allows clinicians to make a diagnosis of ADHD even if the child fails to manifest all the required symptoms, whereas the ICD10 classification does not. Lahey et al. ( 2006) reported that only 26% of children with a diagnosis of ADHD according to the DSM-IV system actually met the ICD10 criteria, and Lee et al. ( 2008) reported an even lower rate, at only 11%. Accordingly, studies based on the DSM-IV indicate prevalence rates of ADHD between 5% and 12%, compared with ICD10 rates between 0.5% and 5% (Skounti et al. 2007). Therefore, it is clear that off-label use is difficult to define and identify. Accordingly, we chose age limit as a criterion to define off-label use, as this parameter is independent of the different classification systems.

To date, the new, updated DSM-V classification (American Psychiatric Association 2013) has not yet been integrated into the regulatory indications for the use of methylphenidate. This new classification widens the diagnostic criteria for ADHD as compared to the previous version, by requiring only that symptoms appear before the age of 12 years, instead of 6 years. The patient must present signs of inattention and/or hyperactivity. Each item is characterised by 9 criteria, of which 6 must be present in children and only 5 in adults (>17 years) in order for the diagnosis to be made. In practical terms therefore, the population potentially meeting these new criteria is considerably widened, and if these new criteria are integrated into the prescription indications for methylphenidate, then off-label use will become exceedingly difficult to detect. Such a wide potential target population for methylphenidate use is cause for concern. Off-label use of the drug in the adult population will not be resolved by uptake of the new definition into the labelling and product characteristics. Indeed, our study shows that there is considerable use of methylphenidate in indications other than ADHD.

Despite being on the market for almost 60 years, the tolerance of methylphenidate has never been studied in detail. One study from the UK Medicines and Healthcare products Regulatory Agency, which collects suspected drug reactions in patients aged <17 years, reported that, excluding vaccines, methylphenidate was the medication most commonly associated with adverse reactions in children (Hawcutt et al. 2012). The long term safety of methylphenidate use is poorly documented. For example, the long term implications of continuous treatment from childhood to adulthood in terms of height, or long term changes in the brain are unknown. The cardiotoxicity of methylphenidate, in terms of tachycardia, hypertension, or potential valvulopathy, remains debated in the literature. Indeed, methylphenidate has been shown *in vitro* to bind to the 5-hydroxytryptamine (HT) receptor 2B (Markowitz et al. 2006), and activation of these receptors has previously been shown to be involved in the development of valvular heart disease (Andrejak and Tribouilloy 2013; Huang et al. 2009). While binding affinity has been demonstrated, the pharmacological effects (notably agonist or antagonist activity) must be elucidated. This potentially harmful effect warrants close surveillance. Preliminary findings suggest that methylphenidate exerts binding activity as an agonist of 5HT receptor 1A (Markowitz et al. 2009).

Pulmonary hypertension has also previously been alleged to result from treatment with CNS stimulant drugs. In 1972, Lewman ( 1972) published a case report of death occurring in a patient aged 30 years, who was a drug addict taking barbiturates, amphetamines, LSD and marijuana for 7 years. Under methadone treatment, the patient injected methylphenidate intravenously (Ritaline^®^), at a rate of 5 tablets per day for 7 months. The patient developed pulmonary hypertension and subsequently died, and significant pulmonary granulomatosis was observed at autopsy. More recently, Karaman et al. ( 2010) similarly reported pulmonary hypertension occurring in a patient aged 15 years, with clinical signs appearing only four days after initiation of methylphenidate therapy. Classically, methylphenidate is classed among the drugs likely to induce cardiomyopathies (Figueredo 2011; Henderson and Fischer 1995), with numerous reports published in the literature (Dadfarmay and Dixon 2009; Fischer and Baner 1977; Nymark et al. 2008; Tollofsrud and Hoel 2006).

A recent report by Schelleman et al. ( 2012) found a 1.8-fold increase in the risk of sudden death or ventricular arrhythmia, and a 1.7-fold increase in the risk of all-cause death in almost 44,000 new users of methylphenidate. However, the indications for treatment were not reported, and patients were only followed for 60 days. In a retrospective cohort of >800,000 adults aged 25 to 64 years, Habel et al. ( 2011) failed to find any increase in cardiovascular risk in users of ADHD medications (methylphenidate, amphetamine and atomoxetine) as compared to matched non-user controls. However, a meta-analysis by Mick et al. ( 2013) reported a statistically significant increased risk of high (>90 bmp) resting heart rate in adult ADHD patients treated with CNS stimulants, and these findings have been confirmed in recent studies (Vitiello et al. 2012; Ginsberg and Lindefors 2012). In paediatric populations, several reports have failed to observe any increased risk of cardiovascular events with methylphenidate in children and youths treated for ADHD (Schelleman et al. 2011; Winterstein et al. 2012; Olfson et al. 2012).

Results are globally quite reassuring, despite individual reports of negative effects in adults (Schelleman et al. 2012; Gould et al. 2009). In addition, 3 cases of heart failure in young adults have recently been reported (Wikstrom et al. 2012). Methodological biases are present in many of the studies reported heretofore, including short follow-up, high rate of patients lost to follow-up, lack of observational data from real-life practice (Molina et al. 2009; Wilens et al. 2003; Fredriksen et al. 2013).

Fredriksen et al. ( 2013) performed a meta-analysis of long-term studies (8 studies in total) with follow-up of minimum 6 months, and maximum 1 year. The most recent study, by Ginsberg and Lindefors ( 2012) was performed in 30 prison inmates with follow-up of 52 weeks. This study confirmed an increase in systolic and diastolic blood pressure under treatment (systolic blood pressure +21 mmHg and diastolic blood pressure +11 mmHg).

The lack of pharmacoepidemiological data on the effects of long-term administration of methylphenidate, in terms of cardiovascular or cerebrovascular risk, impact on growth, risk of cancer, or other risks, is an issue that has often been raised in recent years.

The risk of sudden death in children and adolescents is very low, underlining the need for studies to encompass very large sample sizes.

A case–control study performed by Gould et al. ( 2009) on mortality data from 1985–1996 in the USA among children aged 7 to 19 years of age, showed an association between the use of psychostimulants and sudden death in children and adolescents, with a relative risk of 7. However, the methodology of this study has come in for some criticism.

Data from the WHO for the period 1999–2003 report the occurrence of 25 sudden deaths under psychostimulants, of which 8 were with methylphenidate (7 children, 1 adult) (Wilens et al. 2006).

On 1 November 2011, the FDA published a statement for the general public emphasizing the absence of any relation between the use of psychostimulant drugs for the treatment of ADHD, and the appearance of adverse cardiovascular effects. A study by Cooper et al. ( 2011) that included 1,200,438 children and young adults aged 2 to 24 years undergoing treatment, corresponding to a follow-up of 2,579,104 person-years, reported a rate of 3.1 serious cardiovascular events per 100,000 patient-years. The authors concluded that there was no evidence that the use of ADHD drugs was associated with an increased risk of serious cardiovascular events. The methods and conclusions of this study are at odds with those of the study by Gould et al. ( 2009) showing an association between use of psychostimulants and risk of sudden death in children and adolescents, hence the call from the FDA to adhere strictly to prescription recommendations.

Through its amphetamine-like properties, methylphenidate also exerts anorexigenic effects. In the UK pharmacovigilance database, anorexia is the most frequently reported side effect in children treated for ADHD, representing 34% of all reports (Tobaiqy et al. 2011). These findings confirm reports from the WHO’s Vigibase database indicating that in the 2–11 year age group, ADHD drugs account for 14% of reports, with anorexia cited most often (Star et al. 2011). Non-medical use of methylphenidate in this context should be monitored, as it is possible that some subjects take it to induce weight loss. The amphetamine-like effect is primarily expressed as increased aggression, which is the most frequently reported adverse effect in the 12–17 year age group (Star et al. 2011; Harty et al. 2009).

Methylphenidate has also been shown to have teratogenic effects in animal studies, with reported anomalies of the skeleton, including spina bifida in rabbits, and behavioural disorders in the progeniture of mice treated during pregnancy with 4 times the maximum recommended dose for humans (Chapin 1997; McFadyen-Leussis et al. 2004). Methylphenidate administration in a rodent model of ADHD showed that treatment in adolescent rats enhanced cocaine self-administration during adulthood (Somkuwar et al. 2013). The effects of methylphenidate on the reproductive system are unclear. One study in an animal model found a reduction in testosterone levels in male mice, associated with a reduction in the number of Leydig cells (Fazelipour et al. 2012). In the absence of robust assessments of the teratogenic potential of methylphenidate, prescription its prescription in pregnant women should be contraindicated.

Although ADHD has been recognised as a distinct pathological entity in children since the 19th century, it was only described in adults in the 1970s (Wood et al. 1976), providing the impetus for wider methylphenidate use in adults.

The definition of adult ADHD is the subject of some controversy, with various studies have attempted to describe the persistence of ADHD symptoms in adolescence and adulthood (Barkley et al. 2002; Kessler et al. 2005a, [b], 2006). Several authors purport that adults can present ADHD (Davidson 2008; Faraone et al. 2004; Newcorn et al. 2007; Spencer et al. 1994, 1995) and the diagnosis is associated with a prior history of ADHD in childhood. The diagnosis is based on the same DSM-IV criteria that are applied in children (Biederman et al. 2006; Faraone and Biederman 2005), thus rendering diagnosis difficult. Various diagnostic scores are under study for the specific diagnosis of adult ADHD, such as the Copeland Symptom Cheklist for Adult Attention Deficit Disorders, Wechsler Adult Intelligence Scale, ADHD Self-report Scale-version 1.1 (ASRS-v1.1), Current Symptoms Scale, Wender Utah Rating Scale (WUTS), Brown Attention-Deficit Disorder Rating Scale for Adults (Davidson 2008), as well as neuropsychological tests focusing on attention and learning (Fargason and Ford 1994). The myriad scales available for the diagnosis of ADHD in adults reflect the difficulty physicians encounter in this context (Riccio et al. 2005). It should be noted that, to date, methylphenidate is not approved for the treatment of ADHD in adult populations in France or elsewhere.

## Conclusion

Despite restricted conditions for the prescription of methylphenidate in France, we observed a progressive increase in the number of spontaneous reports of ADRs, with a high proportion of off-label use, in a national pharmacovigilance database. In half the reports where the use was considered to be off-label, the ADR was classed as serious. The widespread use of methylphenidate in adults is a cause for concern. The diagnosis of adult ADHD remains imprecise, and methylphenidate is not indicated in this context at the present time. The profile of side effects appears to differ between adult and paediatric populations, with greater risk of misuse, increased doses, probable cardiovascular effects and disinhibition in adult users. The use of methylphenidate in women aged >18 years incurs a risk of exposition during pregnancy, with potential teratogenic effects. In view of the numerous questions that remain unanswered regarding tolerance of this drug in the long term, its use should be strictly reserved to the approved indications, with evaluation of the risk-benefit ratio on a case-by-case basis. Closely monitoring of methylphenidate prescription practices by the regulatory health authorities is warranted.

Despite being on the market for several decades, the tolerance and mechanisms of action of methylphenidate are still under study. Additional long-term exposures and independent clinical studies are necessary to establish the long-term profile safety of methylphenidate.
